# Adsorption on Ligand-Tethered Nanoparticles

**DOI:** 10.3390/ijms22168810

**Published:** 2021-08-16

**Authors:** Małgorzata Borówko, Tomasz Staszewski

**Affiliations:** Department of Theoretical Chemistry, Institute of Chemical Sciences, Faculty of Chemistry, Maria Curie-Skłodowska University, 20-031 Lublin, Poland; staszewski@umcs.pl

**Keywords:** hairy nanoparticles, adsorption on nanoparticles, nanocarriers, molecular dynamics, computer simulations

## Abstract

We use coarse-grained molecular dynamics simulations to study adsorption on ligand-tethered particles. Nanoparticles with attached flexible and stiff ligands are considered. We discuss how the excess adsorption isotherm, the thickness of the polymer corona, and its morphology depend on the number of ligands, their length, the size of the core, and the interaction parameters. We investigate the adsorption-induced structural transitions of polymer coatings. The behavior of systems involving curved and flat “brushes” is compared.

## 1. Introduction

For many years, polymer brushes have attracted considerable attention due to their interesting properties and many potential applications [1,2,3,4,5,6,7,8,9,10,11]. The brushes can be synthesized on planar surfaces as well as on nanoparticles. In such polymer coatings, ligands are chemically attached to a substrate. The unique structure of the tethered polymer layers makes them very interesting from a purely practical point of view. Due to the high mobility of the chains, their configurations can change with an environmental stimuli, such as the pH, temperature, and salt concentration [2,3]. These coatings are also thicker and mechanically more stable than physically adsorbed monolayers [1].

Recent advances in polymer chemistry have enabled the synthesis of polymer brushes with well-defined structures and tunable properties [1]. Similarly, developments in nanomaterial chemistry have produced polymer-tethered (hairy) nanoparticles with narrow size distributions and controllable physicochemical characteristics of polymer coronas [2]. These hybrid nanoparticles offer a novel platform for the application of brush systems in diverse fields, such as the production of nanocomposites [4], sensing [5], biotechnology, and biomedicine [6]. The particle-based polymer brush systems can be applied as drug delivery vehicles and as carrier materials for proteins or enzymes [7,8,9,12,13,14].

These drug delivery systems are of great interest for the treatment of cancer and other diseases [7,8,9,12]. Many kinds of drug carriers have been developed [7], including various hairy nanoparticles. Their polymer coatings prevent aggregation and facilitate conjunction with drugs and compounds that can protect the carrier against recognition by the immune system. They can deliver drugs on-site, resulting in higher drug transport efficiency, lower dosages required, and reduced side effects. Hairy nanoparticles can be exploited not only for drug delivery but also for monitoring the treatment’s effects or enhancing its efficiency [7,9].

Polymer-tethered magnetic nanoparticles can serve as contrast agents in magnetic resonance imaging [13]. The delivery of drug molecules to the target place consists of three steps, namely the loading of the molecules to a carrier, their transport in the blood, and the triggered release of drugs from a carrier in response to a stimulus encountered on entry into the diseased tissue [7]. Understanding these processes is essential for the rational design of drug delivery systems and other applications of polymer-based carriers.

The great variety of polymeric coatings makes hairy nanoparticles amenable to various loading strategies. The active molecules can form chemical bonds with functional groups in polymer chains or accumulate in the brush via physical adsorption [7]. On the other hand, polymer brushes can be designed to eliminate or significantly reduce the adsorption of biomolecules onto surfaces [10,11]. Hence, it is important to develop insight into the interactions between molecules or colloidal particles and polymer brushes to develop approaches to control the adsorption characteristics.

Regardless of the purely practical motivations for investigating polymer-tethered layers, these systems are extremely interesting from a fundamental point of view. Therefore, polymer brushes on flat substrates were extensively studied using different theoretical methods, including scaling theory, the self-consistent field theory, and the density functional theory [15,16,17,18,19]. Most of the research focused on modeling the morphology of brushes by changing parameters, such as the type of tethers, their lengths, the grafting density, the interactions of chains with the environment, and the temperature. The results were summarized in several reviews [15,16,19].

The morphology of brushes on curved surfaces was less extensively studied. Some works used the similarity between polymer-tethered particles and star polymers [20,21]. A mean-field theory for star polymers was developed by Daoud and Cotton [22]. This theory predicted more stretched chain conformations in the vicinity of the surface due to excluded volume effects and relaxed chain conformations for the peripheral region of the star. Ohno et al. [20,21] extended the Daoud–Cotton model to the polymer-tethered spherical particles of different sizes.

In contrast to flat surfaces, particle curvature implies that the area per chain increases with distance from the surface, and thus the outer ends of the chain have more space. The self-consistent field model and the scaling theory were used to explore the properties of finite chains tethered on curved interfaces [23,24]. Williams and Zhulina [24] described polymer brushes at spherical and cylindrical surfaces immersed in implicit solvents. They analyzed the effects of the surface curvature and the solvent quality of the brush architecture. In turn, Lo Verso et al. [25] used molecular dynamics simulations and density functional theory to study polymers end-grafted to spherical nanoparticles under good solvent conditions.

The behavior of various molecules and particles near brushes was also the subject of theoretical considerations. Most of this research has focused on particles near flat surfaces modified with tethered chains. The adsorption of small molecules [26,27,28,29,30], polymers [31,32,33,34], peptides, proteins [35,36,37,38,39] and Janus particles [40,41] on the flat brushes was interpreted in the framework of different approaches. It has been shown how the adsorption depends on the grafting density, the chain length, and interactions between all species [19,26,27,28,29,30,31,32,33,34]. However, in the case of hairy nanoparticles, research has focused on their interactions with small molecules [42,43,44,45,46] or proteins [47,48,49].

Grest’s group [42,43,44,45,46] used fully atomistic molecular dynamic simulations of spherical particles modified with various ligands solvated in water and organic solvents and on interfaces [46]. Quite recently, Chew et al. [48] studied the hydrophobicity of monolayer-protected gold nanoparticles using atomistic molecular dynamics simulations. They calculated local hydration free energies at the nanoparticle–water interface and found that these energies were correlated with the preferential binding of propane as a representative hydrophobic probe molecule.

It is difficult to theoretically predict the amount of adsorption on hairy particles. The reason lies mainly in the complexity of the problem. Hairy nanoparticles can be treated as “living adsorbents”; their internal morphology changes in a response to interaction with the environment. In addition, the adsorption of fluid molecules induces the reconfiguration of tethered chains [43,45,48,50]. The chains attached to a curved surface are more mobile than those tethered on a flat substrate.

Thus, for hairy particles, these effects become stronger [19,20,21,23]. Furthermore, nanoparticles are typically dispersed in a solvent, and they can aggregate due to interactions between molecules adsorbed on different hairy particles [50,51]. There are only a few theoretical works devoted to a description of the adsorption on hairy nanoparticles [50,52] or hairy vesicles [53].

Adsorption on hairy nanoparticles was also a subject of experimental studies [52,54,55,56,57]. For example, Ballauff et al. [52,54,55] analyzed the impact of several parameters on the adsorption on polyelectrolyte layers. However, Snytska’s group [56,57] studied the effects of grafting density on the properties of polymer brushes prepared on flat and on colloidal particle substrates. By changing the grafting density, they tuned the properties of the carrier material, such as swelling, charge, adhesion, and the adsorption of enzyme on grafted brushes. The adsorption of albumin on magnetic submicrospheres with a hairy core-shell structure was also measured [58]. Wang et al. [51] presented the results of research concerning the co-immobilization and separation of proteins using particles modified with polyelectrolytes.

All the above-mentioned studies demonstrated the complexity of the processes underlying the loading of molecules onto nanoparticle-based carriers. Therefore, a thorough description of the adsorption on hairy nanoparticles remains an open challenge. There is still a lack of systematic investigations concerning the correlation between the properties of polymer corona and the efficiency of adsorption on hairy particles and the spatial distribution of adsorbed molecules around the cores.

In this work, we study an idealized coarse-grained model for the adsorption of spherical molecules on polymer-tethered particles by molecular dynamics simulations. Our main purpose is to give a quantitative description of adsorption onto hairy nanoparticles under different conditions and to examine the behavior of spherical brushes in the presence of small particles. We change such parameters as the core diameter, grafting density, length of chains, their flexibility, interactions between all moieties, and the density of adsorbed particles. In this way, we can modulate the thickness of the polymer corona, its internal morphology, and the adsorption of fluid molecules onto a hairy nanoparticle. We want to capture basic factors determining the adsorption properties of hairy particles. Some results are compared with their planar counterparts.

We consider nanoparticles with rather short ligands. The behavior of such nanoparticles is relatively less explored compared with those with very long chains. However, these systems are very interesting from a purely cognitive point of view. Additionally, due to savings in computation time, it is possible to scan a wider range of parameter space. This allows us to capture basic factors determining the adsorption properties of hairy particles.

## 2. Model and Simulation Methodology

We consider a hairy particle immersed in a fluid consisting of spherical particles *P* (molecules or small colloids). For computational efficiency, we coarse-grained our system to reduce the number of “atoms” required. A single tethered nanoparticle was modeled as a spherical core with attached *f* chain molecules. The core diameter equals $\sigma_{c}$, and the particles *P* have the diameter $\sigma_{P}$. Each chain consists of *M* tangentially jointed spherical segments of identical diameters $\sigma_{s}$. The chain connectivity is assured by the harmonic segment–segment potentials

(1) $$u_{ss}^{\left(b\right)}=k_{ss}{\left(r-\sigma_{s}\right)}^{2},$$

where *r* is the distance between segments. The first segment of each chain is rigidly fixed to the core at a randomly chosen point on its surface (at the distance $\sigma_{cs}=0.5\left(\sigma_{c}+\sigma_{s}\right)$). We assume that the chains are perfectly flexible, and, in each chain, segments are freely connected (model M1).

All the spherical entities interact via the shifted-force Lennard–Jones potential [59]

(2) $$u^{\left(ij\right)}=\left\{\begin{array}{cc} 4\varepsilon_{ij}\left[{\left(\sigma_{ij}/r\right)}^{12}-{\left(\sigma_{ij}/r\right)}^{6}\;\right]+\Delta u^{\left(ij\right)}\left(r\right), & \;\;\;r<r_{cut}^{\left(ij\right)},\\ 0, & \;\;\;\mathrm{otherwise}, \end{array}\right.$$

where

(3) $$\Delta u^{\left(ij\right)}\left(r\right)=-\left(r-r_{cut}^{\left(ij\right)}\right)\partial u^{\left(ij\right)}\left(r_{cut}^{\left(ij\right)}\right)/\partial r,$$

In the above $r_{cut}^{\left(ij\right)}$ denotes the cutoff distance, $\sigma_{ij}=0.5\left(\sigma_{i}+\sigma_{j}\right)$ (i,j=c,s,P) and $\varepsilon_{ij}$ is the parameter characterizing interaction strengths between spherical species *i* and *j*. The indices “*c*”, “*s*”, and “*P*” correspond to the cores, the chain segments, and the small particles, respectively. All particles are dispersed in an implicit solvent that determines the strength of interactions between them.

We also study hedgehog-like particles with rigid ligands (model M2). Furthermore, we consider also the adsorption of the particles *P* on a planar surface modified with end-tethered chains using a model analogous to that described above. In this case, however, the solid surface interacts with all particles except the ligand’s bonding segments via the hard-wall potential.

We introduce standard units. The diameter of segments is the distance unit, $\sigma_{s}=\sigma$, and the segment–segment energy parameter, $\varepsilon_{ss}=\varepsilon$, is the energy unit. The mass of a single segment is the mass unity, $m_{s}=m$. The basic unit of time is $\tau =\sigma \sqrt{\left(\varepsilon /m\right)}$. The reduced temperature is $T^{*}=k_{B}T/\varepsilon$.

In this study, particles *P* and chain segments have the same mass, *m*. The spring constant of the binding potential, $k_{ss}=1000\varepsilon /\sigma^{2}$.

In our simulations, core–core, core–chain, and particle–core interactions are purely repulsive, while particle–chain (particle–segment) interactions were attractive. In some simulations, we assume that also segment–segment and particle–particle are attractive. To switch on or switch off attractive interactions, we use the cutoff distance. For attractive interactions, $r_{cut}^{\left(ij\right)}=2.5\sigma_{ij}$, while, for repulsive interactions, $r_{cut}^{\left(ij\right)}=\sigma_{ij}$. The grafting density is defined as $\rho_{gr}=f/A$, where A is the area of the substrate.

Molecular dynamics (MD) simulations were performed using the LAMMPS classical MD code [60,61]. The Nose–Hoover thermostat was used to regulate the temperature. The reduced temperature was $T^{*}=k_{B}T/\varepsilon =1$ for all the results reported here. Two series of simulations were carried out, for the hairy nanoparticles and the planar brushes.

In the first case, a cubic simulation box was used with the nanoparticle’s core fixed at the center of the simulation cell. The box size varied from L=57σ to L=107σ depending on the system studied. In the case of bigger cores and longer tethers, bigger boxes were used. Standard periodic boundary conditions were introduced in all directions.

The first segment of each tethered ligand was fixed at a distance $\sigma_{cs}$ away from the center of the nanoparticle. These binding beads are randomly distributed on a spherical surface. All the tethering points were then kept fixed thereafter. The simulation cell always had a size capable of accommodating each hairy particle with stretched ligands, the cloud of adsorbed particles, and a sufficiently large part of the bulk fluid. The sphere inscribed (with the radius R′=L/2) in the cube was treated as a virtual adsorption system submerged in the bulk fluid.

A planar brush was simulated in a rectangular box with sizes $L_{x}=L_{y}$ and $L_{z}$, in directions *x*, *y*, *z*, respectively. The wall located at z=0 was covered by the ligands, while the wall at $z=L_{z}$ was a bare, hard wall. The ligands were attached to the flat surface in the same way as to the nanoparticle. The distance between these walls was large enough to ensure the existence of the region of a uniform fluid in the middle part of the cell. The system is periodic in the *x* and *y* directions. The distance $L_{z}$ ranged from 40 to 80. In the majority of the runs, the box dimension $L_{x}$ was 100. In this case, the whole simulation cell is considered as the adsorption system.

The spherical particles, *P*, were randomly inserted into the system until their desired density, $\rho_{0}$ was reached. The initial density of the particles *P* is defined as $\rho_{0}=N/V$, where *N* is the number of the particles and *V* is the cell’s volume.

Simulations were performed for several dozen sets of parameters characterizing the nanoparticle, for two values of diameter $\sigma_{c}=2\sigma$ and 4σ, different lengths of ligands M=5,10,15,20,30, and numbers of tethered chain f=5,10,20,30. In each case, the density of particles *P* varied from $\rho_{0}=0.0001$ to $\rho_{0}=0.3$. For selected parameters, simulations were also carried out for the planar surfaces with attached ligands.

To obtain proper equilibrium, we used very long simulations, simulations with different starting configurations for each system, and alternating heating and cooling of the system. We equilibrated the system for at least $10^{8}$ time steps until its total energy reached a constant level, at which it fluctuated around a mean value. The production runs were for at least $10^{7}$ time steps. At the time, data were saved after every 1000 time steps and used for the evaluation of the local densities of chain segments, $\rho_{s}\left(z\right)$, and the fluid particles *P*, ρ(r), where *r* was the distance between the core center and a segment or a particle. The results presented here were averaged over at least five statistically independent systems. The process was the same for nanoparticles and planar surfaces.

## 3. Results and Discussion

### 3.1. Description of the Studied Systems

We studied the adsorption of small particles on different hairy particles and the flat surfaces modified with tethered chains.

As already mentioned, the simulations were carried for the inert cores. The soft repulsive core–segment and core–particle interactions were assumed. All energy parameters $\varepsilon_{ij}=\varepsilon$ (i,j=c,s,P) except for the particle–segment energy parameter, which is varied ($\varepsilon_{Ps}=1.5\varepsilon$ and 3.0ε). The motivation for choosing these values originates from the fact that similar parameters have already been used for planar brushes [27,28,41] and hairy particles [50]. In the framework of the implicit solvent model, these parameters correspond to the good solvent conditions [19]. Thus, the tethered chains are solvophilic.

We discuss here the results obtained for the nanoparticles with attached flexible (model M1) or rigid ligands (model M2). Most simulations were performed for the systems in which only particle–segment interactions are attractive. However, some simulations were also carried out for the case of attractive segment–segment and particle–particle interactions ( models M1a, M2a). Table 1 shows the types of interactions in different models.

We begin with a detailed analysis of the behavior of the M1-systems. Then, we present selected results for different models.

Our “basic” model (M1) mimics a typical hairy particle with attached flexible chains. For simplicity, only particle–segment models are assumed to be attractive. First, we study the system behavior for relatively low interactions and set $\varepsilon_{Ps}=1.5\varepsilon$. In this case, we study the cores of two sizes ($\sigma_{c}=4\sigma$, 2σ) and the corresponding flat substrate. Different numbers of attached chains (*f*) and different chain lengths (*M*) are assumed.

### 3.2. Excess Adsorption Isotherms

A basic measure of adsorption is the excess adsorption [27,50]. For adsorption on a single spherical hairy particle, the excess adsorption (per particle) is defined as

(4) $$\Gamma =4\pi \int r^{2}\left(\rho \left(r\right)-\rho_{b}\right)dr,$$

where $\rho_{b}$ is the reduced density of molecules *P* in the bulk phase. This value is determined from the analysis of the density profiles. Far away from the center of the core, its impact diminishes, and the density achieves a constant value, $\rho_{b}$ [27,50].

We normalize the excess adsorption in the following way

(5) $$\Gamma^{*}=\Gamma /A,$$

where A is the surface area. For nanoparticles, it is the area of the core surface, $A=\pi (\sigma_{c}^{2})$.

In the case of a flat surface, the excess adsorption (per a unit of surface area) is calculated from the well-known equation [27]

(6) $$\Gamma^{*}=\int \left(\rho \left(z\right)-\rho_{b}\right)dz,$$

where *z* is a distance from the surface. Clearly, Equation (5) becomes a form of Equation (6) for $R_{c}\to \infty$.

The excess adsorption can be also estimated from the following equation

(7) $$\Gamma =V\prime (\rho_{0}-\rho_{b}),$$

where V′ is the volume of the virtual adsorption system, $V\prime =V_{k}=\pi L^{3}/6$ for the particle, and V′=V for the flat surface. The last equation is used for the experimental measurement of adsorption. In some cases, to analyze the simulation results, we used both of these equations, and the obtained values were consistent.

**Figure 1:**
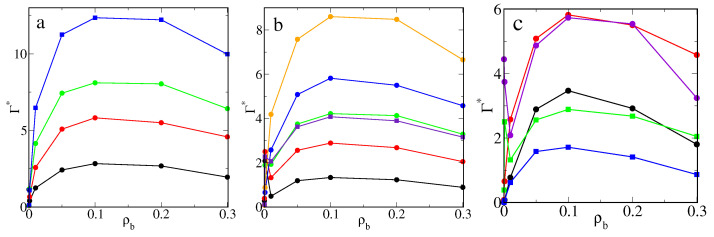
Excess adsorption isotherms for the model M1 and $\varepsilon_{Ps}=1.5\varepsilon$: (**a**) the hairy particle, $\sigma_{c}=4\sigma$, M=20 (circles): f=10 (black), 20 (red), 30 (green), and M=30, f=30 (blue squares), (**b**) the hairy particle, $\sigma_{c}=4\sigma$, f=20 (circles): M=5 (black), 10 (red), 15 (green), 20 (blue), 30 (yellow), and f=30, M=10 (violet squares), (**c**) M=20, $\rho_{gr}=0.398$ (circles): the flat substrate, f=20 (black), the hairy particle, $\sigma_{c}=4\sigma$, f=20 (red), the hairy particle, $\sigma_{c}=2\sigma$, f=5 (violet), and f=20, M=10 (squares): the flat substrate (blue), the hairy particle, $\sigma_{c}=4\sigma$ (green). Symbols correspond to the simulation points. Lines serve as a guide to the eye.

In Figure 1, we show the excess adsorption isotherms of particles P on different hairy particles as well as on flat brushes. Parts a and b present the results for the core of diameter $\sigma_{c}=4\sigma$ and different numbers of attached chains (a) and their lengths (b). Usually, when density $\rho_{b}$ increases, the excess adsorption rapidly rises, reaches its maximum, and begins to slowly decline. However, for short chains or very small numbers of ligands, an atypical course of excess adsorption isotherms is seen.

After an initial rapid increase, the adsorption drops to a deep minimum and begins to rise again. For higher densities, $\rho_{b}$, the excess isotherms have a usual shape. The effect of the “superadsorption” at low densities will be discussed later. An increase in the number of ligands and their length cause an increase in the adsorption. This is a consequence of the assumed model. As the total number of attractive segments increases, the number of potentially available “adsorption sites” also increases.

In Figure 1c, the effect of the surface curvature is shown. We compare the excess adsorption isotherms for brushes with the same ligands (M=20) and the same grafting density ($\rho_{gr}=0.398$). The chains are attached to the bigger ($\sigma_{c}=4\sigma$, red line), the smaller core ($\sigma_{c}=2\sigma$, violet line), and the flat substrate (black line). The impact of the curvature on the magnitude of adsorption is visible. First, the adsorption (per unit of area) on a flat brush is considerably smaller than on spherical hairy particles. Due to geometrical reasons, a volume of “the adsorbed phase” is larger for a spherical brush, and thus the number of adsorbed particles is greater.

Second, the core size also affects the adsorption. However, this effect is significant only in the case of very low or relatively high bulk densities of adsorbed particles. Note that the local minimum of adsorption at very low density only appears for the smaller core. The number of segments in the polymer layer is much greater for a big core compared to a small one with the same grafting density. Therefore, the excess adsorption on a big particle becomes considerably greater at high densities.

Additionally, in Figure 1c, we present the adsorption isotherms for the same number (f=20) of shorter ligands (M=10) grafted to the flat substrate (black line) and the big core (green line). In the case of flat brushes, an anomaly in excess adsorption does not occur for both longer and shorter chains.

Overall, our simulations show the possibility of controlling adsorption on hairy nanoparticles by changing the core size, the chain length, and the grafting density. For the systems tested here, the adsorption increases as the total number of segments increases. Adsorption on nanoparticles is significantly greater than on flat brushes.

In general, our results are qualitatively consistent with experimental observations [56,57,58]. However, direct comparison is difficult. There is still a lack of systematic experimental investigations concerning the correlation between the adsorption isotherms and the properties of the hairy nanoparticles. One can find the adsorption isotherms measured for one type of nanoparticles [58] or the values of adsorption estimated at one arbitrary chosen fluid density on different nanoparticles [56].

Marschelke et al. [56] investigated the immobilization of laccase from Trametes versicolor onto hairy particles with a shell built of poly-(2-dimethy laminoethyl methacrylate) (PDMAEMA). This study showed that the polymer loading (adsorption) showed a maximum at different enzyme concentrations depending on the grafting density of the brushes. They also presented the dependence of the polymer loading on the grafting density for different enzyme concentrations.

At a certain low concentration, the polymer loading decreased with the increase of the grafting density. In the case of higher concentrations, the loading increased, achieved a maximum, and decreased. In a wide range of higher concentrations, however, the polymer loading increased with the grafting density as in our simulations.

Adsorption isotherms on hairy nanoparticles are usually estimated only for very dilute solutions and densely grafted substrates [58]. Under such conditions, the adsorption monotonically increases with the increase of the initial concentration of adsorbed particles. The same trend is observed at the beginning of “typical” isotherms obtained from our simulations. However, we also obtained isotherms with the additional maximum at low particle concentration. To the best of our knowledge, this “superadsorption” at the ligand-tethered nanoparticles has not been reported thus far.

### 3.3. Thickness of Polymer Layer

Adsorption on a brush strongly depends on its structure. On the other hand, the brush morphology varies during adsorption [48,50]. It is well-known that the configuration assumed by the chains depends on the entropic and enthalpic contributions to the free energy. The chains tend to maximize their configurational entropy by adopting a random-walk configuration [19,23]. Simultaneously, they minimize the potential energy by profitable adsorbed particle–segment contacts. The brush structure follows from the competition between these trends.

The average thickness of the polymer layer reflects, to some degree, its structure. In the case of spherical hairy particles, the thickness of the polymer corona is given by [25,43]

(8) $$H=2(\frac{\int r^{3}\rho_{s}\left(r\right)dr}{\int r^{2}\rho_{s}\left(r\right)dr}-R_{c}).$$

For flat substrates, one can obtain [25]

(9) $$H=2\frac{\int z\rho_{s}\left(z\right)dz}{\int \rho_{s}\left(z\right)dz}$$

We define the relative thickness of the polymer layer as the ratio of the estimated value *H* to the thickness for completely stretched chains, H* = *H*/*M*.

**Figure 2:**
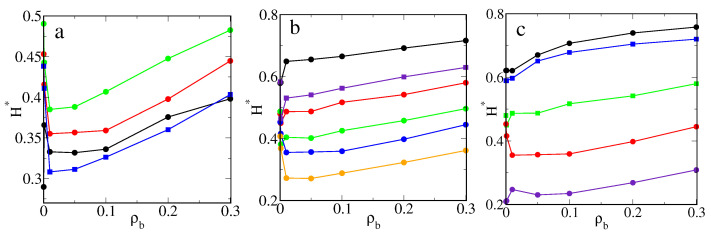
The average thicknesses of the corona for the model M1 and $\varepsilon_{Ps}=1.5\varepsilon$: (**a**) the hairy particle, $\sigma_{c}=4\sigma$, M=20 (circles): f=10 (black), 20 (red), 30 (green), and M=30, f=30 (blue squares), (**b**) the hairy particle, $\sigma_{c}=4\sigma$, f=20 (circles): M=5 (black), 10 (red), 15 (green), 20 (blue), 30 (yellow), and f=30, M=10 (violet squares), (**c**) M=20 and $\rho_{gr}=0.398$ (circles): the flat substrate, f=20 (black), the hairy particle, $\sigma_{c}=4\sigma$, f=20 (red), the hairy particle, $\sigma_{c}=2\sigma$, f=5 (violet), and f=20, M=10 (squares): the flat substrate (blue), the hairy particle, $\sigma_{c}=4\sigma$ (green). Symbols correspond to simulation points. Lines serve as a guide to the eye.

Figure 2 shows the relative average thickness of the bonded phase as a function of the bulk density ($\rho_{b}$) for different model systems. In part a, the thicknesses $H^{*}$ are plotted for rather long chains (M=20) and different numbers of ligands. One can see that the thickness of the polymer corona was strictly correlated with the excess adsorption isotherms. For f>10, all presented curves, similarly to the corresponding excess adsorption isotherms, have the same shape. As the density increased, the $H^{*}$ fell sharply, then in a narrow region remained almost constant and rose linearly.

At low densities, particles P can deeply penetrate the polymer corona, and their adsorption rapidly increases. The adsorbed particles form bridges between segments and the polymer. An increase in the number of adsorbed particles enhances this effect. However, after reaching a certain threshold density, there is no more room for additional particles and the chains begin to unfold; thus the thickness gradually increases. However, for rather rarely grafted cores with f=10, at extremely low densities, a local maximum is visible (black line). The relative thickness of the polymer corona increased as the number of ligands increased. Due to the effect of the excluded volume, the chains became more stretched.

Figure 2b displays the impact of the chain length on the functions $H^{*}$ vs. $\rho_{b}$ and the fixed number of ligands (f=20). After a sharp minimum at low densities, $H^{*}$ increased linearly. The same was found for the system with a higher grafting density (f=30) and M=10 (violet squares). However, in the region of densities considered, there was no minimum on the curve plotted for very short chains (M=5). The thickness jumped rapidly and then increased linearly. Comparing these curves, we found that an increase of chain length at the same grafting density led to a considerable decrease of the relative corona thickness. This can be explained by increasing the adsorption of particles and the enhancement of the bridging effects.

In part c, the effect of the curvature on the average brush thickness is presented for systems from Figure 1c. At the same grafting density (full circles) the thickness increased with decreasing surface curvature. In the case of the flat surface, the relationship $H^{*}$ vs. $\rho_{b}$ is a slowly increasing function, while, for spherical cores, slight jumps on the curves are visible at very low densities.

Let us briefly discuss the problem of the thickness of polymer-tethered layers. It is well-known that the average height of the flat brush depends mainly on the chain length, the grafting density, and solvent quality [19]. Scaling theories and the self-consistent field theories [19,23,24] predict that the height of the brush increases as the chain length increases; simultaneously, its relative thickness decreases. Thus, our results are qualitatively consistent with theoretical predictions. Similarly, the grafting density increased in accordance with a power-law dependence [19].

Such a relation accurately approximates the data obtained for dense brushes. However, density functional studies [62] showed that the behavior of sparse brushes can be completely different. For very low surface densities of tethered chains, the brush height remained almost constant. In this case, the grafted chains did not practically affect one another, and they assumed unperturbed configurations. For medium-covered substrates, the height fell to a minimum [62]. With a further increase of the grafting density, the brush height increases as in the predicted scaling theories [19]. Although, we also took into account the rather sparse brushes (f=5,10). In all systems considered here, an increase of the grafting density led to the formation of thicker brushes.

The effect of the adsorption of particles on the brush height was much less studied. We analyzed the impact of the fluid density on the brush height using density functional theory [62,63]. Increasing $\rho_{b}$ can both increase or decrease the brush height. The course of the functions *H* vs. $\rho_{b}$ depends on all interactions in the system [63]. The experimental studies confirmed that the brush height can vary in a complex way during the adsorption.

Marschelke et al. [56] found that the thickness of the swollen PDMAEMA brushes was significantly reduced after the immobilization of enzyme, while the opposite effect was observed for dry brushes. However, Minko et al. [64] reported that the adsorption of enzyme on poly(acrylic acid)-modified (PAA) particles caused the increase of polymer brush thickness in the swollen state. Unfortunately, there is no information about the impact of enzyme concentration on the brush thickness.

A decrease in the brush thickness is commonly explained by the strong attractive interactions between segments [19,23,24]. In our model, this mechanism is completely distinct. Particle–segment interactions play a decisive role to cause the adsorption of particles on “chains”. In turn, adsorbed particles influence the chain configurations. The particles form bridges between segments belonging to the same chain and to other chains. Such a bridging effect was observed experimentally [51] The chains wrapped around particles and the polymer corona became more compact. For a sufficiently dense fluid, the adsorbed particles pull segments to the bulk phase so that the height increases. Similar results were obtained for adsorption on nanoparticles with mobile ligands [50].

### 3.4. Structure of Interfacial Layers Formed on Nanoparticles

To obtain a deeper insight into the structure of the polymer layers, we calculated the one-dimensional density profiles of chain segments, $\rho_{s}\left(r\right)$ and the density profiles of adsorbed particles, ρ(r), around the core.

**Figure 3:**
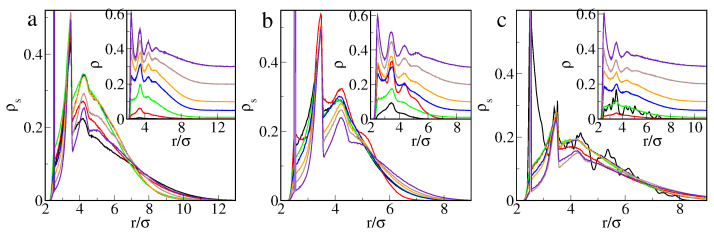
Density profiles of the chain segments and fluid particles (insets) around the core for the model M1 plotted for different initial densities of the fluid $\rho_{0}=0.0001$ (black), 0.001 (red), 0.01 (green), 0.05 (blue), 0.1 (yellow), 0.2 (brown), and 0.3 (violet): (**a**) f=20, M=20, (**b**) f=20, M=10, (**c**) f=10, M=20. Other parameters: $\sigma_{c}=4\sigma$, $\varepsilon_{Ps}=1.5\varepsilon$.

Figure 3 presents the segment density profiles and the density profiles of adsorbed particles on the big nanoparticle (σ=4σ) for different numbers of chains and their lengths, (a) f=20, M=20, (b) f=20, M=10, and (c) f=10, M=20, calculated at different initial densities of particles $\rho_{0}$.

In the segment density profiles for the higher grafting density (Figure 3a,b), one sees three or four peaks. The first peaks correspond to grafted segments that are located directly at r=2.5σ. The pronounced structure is also visible further from the core. The second segments in chains show up as rather sharp peaks at r=3.5σ. The next peaks at r=4.5σ are much wider. Then, the segment density smoothly decreases. The molecules are attracted by the chains, due to which, they penetrate deeply into the polymer layer. In the fluid density profiles, ρ(r), one sees peaks corresponding to “adsorption on” the subsequent layers of segments. The density of the fluid inside the brush is greater than in the bulk phase.

An initial density of the adsorbed molecules affects the structure of the surface layer. In the case of longer ligands, an increase of the density $\rho_{0}$ causes an increase in the density of the particles inside the polymer layer. Such a relation is observed for the “normal” excess adsorption isotherms. For short tethered chains, however, the density profile corresponding to $\rho_{0}=0.001$ (red line) is much greater than that estimated at $\rho_{0}=0.01$ (green line). This reflects the existence of a local maximum at the beginning of the excess adsorption isotherm. At $\rho_{0}=0.001$, three well-pronounced peaks are visible in the density profile of the fluid. In the same part of the brush, the segment density is very high. This suggests the occurrence of a reconfiguration in the polymer corona at this fluid density.

The local maximum at the beginning of excess adsorption isotherm was also found for low grafting density (f=10) and long chains (M=20). Figure 3c depicts the corresponding density profiles. Here, the segment densities in the middle part of the polymer layer were much lower than those for the dense brushes. The segment density was the highest for the lowest density $\rho_{0}$. In the center part of the corona, the local fluid density at $\rho_{0}=0.0001$ (black line) was considerably greater than at $\rho_{0}=0.001$ (red line). The one-dimensional segment density profiles, ρ(r), show the densities averaged on a sphere of radius *r*. They do not show all changes in the shape of the polymer corona.

**Figure 4:**
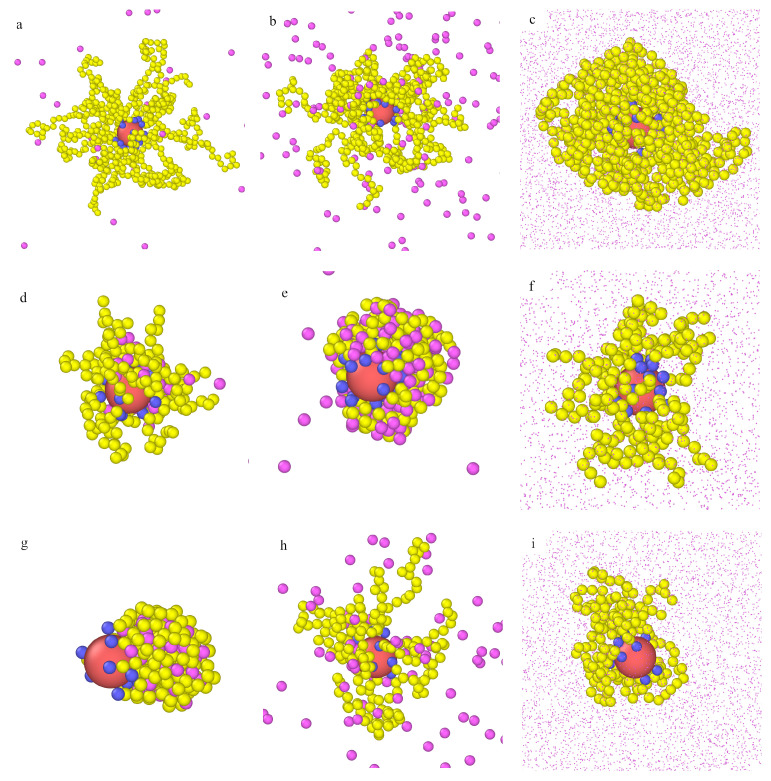
Examples of the equilibrium configurations of hairy particles (model M1, $\varepsilon_{Ps}=1.5\varepsilon$) immersed in fluids of different densities $\rho_{0}=0.0001$ (**a**,**d**,**g**), 0.001 (**b**,**e**,**h**), 0.1 (**c**,**f**,**i**) for f=20 and M=20 (**a**–**c**), f=20 and M=10 (**d**–**f**), and f=10 and M=20 (**g**,**h**,**i**). The red sphere represents the core, and blue spheres correspond to bonding segments, yellow spheres and pink spheres represent the remaining segments and fluid particles P, respectively. For clarity, the particles P are represented by points (size ratios are not kept) in parts (**c**,**f**,**i**).

We monitored the configurations for all studied densities of fluid. The most representative examples for model M1 are presented in Figure 4. In the upper row (a, b, c), the particle with attached long chains (M=30) is presented for different densities of fluid: $\rho_{0}=0.0001$ (a), $\rho_{0}=0.001$ (b), and $\rho_{0}=0.1$ (c). Corresponding configurations for the particles with shorter chains (M=10) are shown in the middle row (c, d, e). In the case of long tethers, at the very low density ($\rho_{0}=0.0001$), the chains are unfolded, and a few particles are arrested inside the polymer layer. The hairy particle has a symmetrical core-shell structure. As more particles are adsorbed, the polymer layer becomes more compact, and, at $\rho_{0}=0.1$, a dense cloud of segments with particles trapped inside is observed.

For the short chains (M=10) at the lowest density $\rho_{0}$, the morphology of the polymer layer is similar to the previous results. However, it changes dramatically at $\rho_{0}=0.001$, and a cone-like structure is found. In this case, adsorbed molecules can easily penetrate the polymer layer, and they accumulate near the chains and form bridges between different chains. The cloud of segments becomes highly asymmetrical. A part of the core remains uncovered.

In the bottom row, the snapshots for f=10 and M=20 are shown. In this case, the cone-like structure appears at the lowest density, as the fluid density increases, the chains stretch and transform into a loose asymmetrical structure. Notice that the total number of segments is the same as for the particle drawn in the middle row. This suggests that the cone-like structure can be stable if there are enough adsorbed particles to stick the segments together. As the density $\rho_{0}$ increases, the excluded volume effects cause gradual extension of the chains, and this characteristic structure can disappear (see Figure 4f).

We also found that an increase of curvature of the core favored the formation of cone-like structures (not shown here). We compared the behavior of particles consisting of cores of different sizes with relatively long attached chains (M=20) and the same grafting densities. For the smaller core, the cone-like structure arose and did not for the larger core.

Overall, our results clearly show that adsorption on hairy nanoparticles particles can significantly change the morphology of the polymer layer. The particles were adsorbed mainly inside the brush, and their presence induced changes in the morphology of the polymer layer. In some cases, asymmetric cone-like structures were found. The asymmetry in the polymer coatings was likely to have a significant effect on the aggregation behavior.

Similar effects were reported by Bolintineanu et al. [45] who carried out atomistic molecular dynamics simulations of different alkanethiol-coated gold nanoparticles solvated in water and decane. In some systems, they found significant local bundling of chains on the nanoparticle surface, which resulted in highly asymmetric coatings [45]. Simulations performed by Chew et al. [48] also showed that ligands tended to form “bundles”, giving rise to anisotropic structures despite homogeneous surface coatings.

In our previous work [50], we presented the adsorption-induced reconfiguration of the polymer corona built of ligands that could freely move on the core surface. We found that, depending on fluid–chain interactions and the fluid density, isolated hairy particles could be classified as core-shell, octopus-like, and corn-like.

Furthermore, Marschelke et al. [56] proved experimentally that the adsorption of the enzyme affects the polymer swelling and, therefore, leads to the changes in the surface morphology, charge, and adhesion performance of the final polymer–enzyme layer.

### 3.5. Structure of Interfacial Layers Formed on a Flat Substrate

To investigate a role of curvature of the substrate, we carried out simulation for brushes formed at a flat surface.

**Figure 5:**
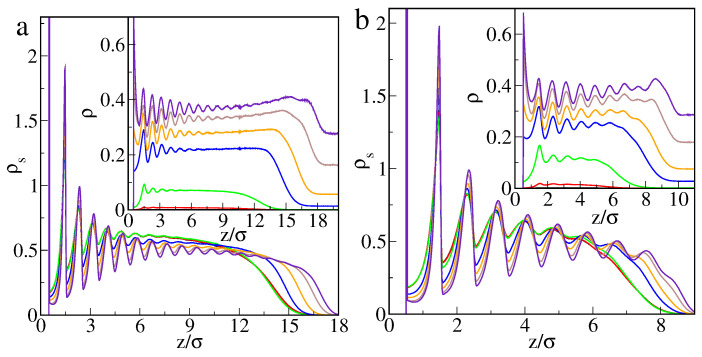
Density profiles of the chain segments and fluid particles (insets) near the flat substrate for the model M1 and different chain lengths: (**a**) M=20, (**b**) M=10 plotted for different initial densities of the fluid $\rho_{0}=0.0001$ (black), 0.001 (red), 0.01 (green), 0.05 (blue), 0.1 (yellow), 0.2 (brown), and 0.3 (violet). Other parameters: f=20, $\varepsilon_{Ps}=1.5\varepsilon$.

Figure 5 illustrates the structure of a bonded layer at the flat surface for the same grafting density as in the case of the hairy particles (see Figure 1c). The segment density profiles have a typical liquid-like structure with the well-pronounced peaks corresponding to subsequent layers of segments. Then, this structure diminishes, and the density of chain segments gradually decreases to zero at the effective brush height. The extent of the layering and the effective brush height increase with increasing the density of adsorbed particles.

In the interior of the polymer layer, the segment density decreases as the fluid density increases. The opposite effect is observed in the outer part of the bonded phase. In the insets, the density profiles of particles are also plotted. The location of peaks in the density profiles of segments and molecules P are the same. As with the hairy particles, molecules are “adsorbed on segments”.

The density of adsorbed particles is high near the surface, then it somewhat decreases and, for high bulk densities, increases again at the brush end. The molecules are adsorbed also “on the brush” (ternary adsorption [15,33]). Similar results have been obtained for the flat surfaces modified with tethered short chains using the functional density theory [26,27,28,29] and molecular dynamics simulations [41].

For geometrical reasons, a direct comparison of the density profiles around the core with those calculated near the flat surface is impossible. However, the polymers grafted to the flat surface formed more segment layers, and they were more stretched. This polymer layer was more compact. In contrast to the hairy particles, on flat surfaces, ternary adsorption was found. Particles penetrated less into such a dense polymer layer and accumulated at its outer part and above it.

**Figure 6:**
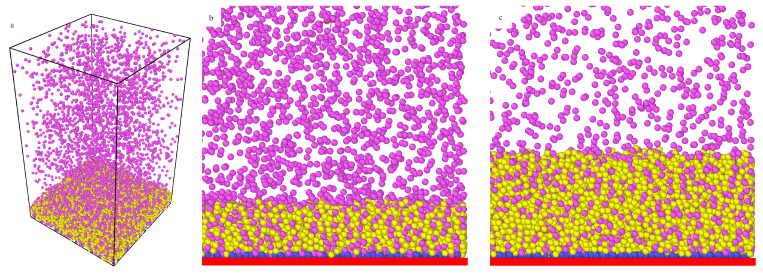
Examples of the equilibrium configurations of the system (M1) involving flat surfaces modified with chains of different lengths: M=10 (**a**,**b**) and M=20. Other parameters: $\rho_{gr}=0.398$, $\varepsilon_{Ps}=1.5\varepsilon$. The red line represents the flat substrate. Blue spheres correspond to bonding segments, yellow spheres and pink spheres represent the remaining segments and fluid particles P, respectively. In parts (**b**,**c**), sideways views of simulation boxes are shown.

In Figure 6, we show examples of configurations of flat brushes immersed in a compatible fluid. The grafting density was the same as in the case of the larger core with attached f=20 chains. In part a, the whole simulation box is shown for M=10. The side sections of the systems are also presented for M=10 (b) and M=20 (c). We see that the adsorption on the layer consisting of a longer chain was considerably greater. This was manifested by considerably smaller density in the bulk phase. Ternary adsorption is clearly visible in the snapshots. One can see that there was less available space between chains than in the case of the hairy particles.

Our study proves that the curvature of the substrate influenced the adsorption and structural properties of the brushes. Experimental investigations of Marschelke et al. [56,57] showed that there was no direct transferability of the results received from planar to curved substrates. Our simulations confirm their conclusion.

### 3.6. Other Models for Adsorption on Ligand-Tethered Nanoparticles

As already mentioned, we also performed a few simulations for models M1a, M2, and M2a for σ=4σ, M=10, and f=20. The results were compared to those obtained for the same geometrical parameters and the basic model M1. We show how the adsorption depended on the degree of flexibility of the ligands, the strength of interactions between the particle and ligand segments, and attractive interactions between the adsorbed particles and between segments.

**Figure 7:**
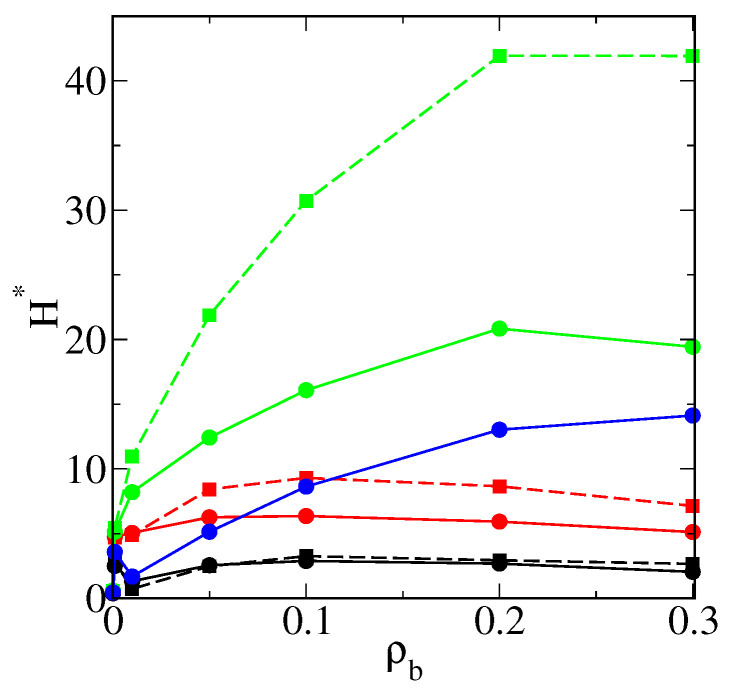
Excess adsorption isotherms on the particle with attached flexible (circles, solid lines) and rigid ligands (squares, dashed lines) for different models of adsorption: models M1 and M2 with ε=1.5ε (black), models M1 and M2 with ε=3.0ε (red), models M1a and M2a with ε=3.0ε (green), and model M1a with ε=1.5ε (blue). Other parameters: f=20, M=10. Symbols correspond to simulation points. Lines serve as a guide to the eye.

In Figure 7, excess adsorption isotherms for different model systems are shown. As one can predict, the adsorption was greater for stronger particle–segment interactions. Increasing the energy parameter ε can cause the disappearance of the local maximum at low density in the excess adsorption isotherm (compare the solid blue and green lines).

The attractive particle–particle and segment–segment interactions intensified the adsorption (compare the red and green lines or black and blue lines). Adsorbed particles additionally attract others, and a wider “adsorption layer” is formed. This effect is particularly visible for $\varepsilon_{Ps}=1.5\varepsilon$ (blue solid line) at high bulk densities. Moreover, it can be seen that, for rigid ligands, adsorption is always greater than for flexible tethered chains.

This effect is minor for $\varepsilon_{Ps}=1.5\varepsilon$ (black lines) and clearly visible for stronger particle–segment interactions, $\varepsilon_{Ps}=3.0\varepsilon$, (red lines). The difference in the behavior of particles with rigid and flexible ligands became much more significant for attractive particle–particle and segment–segment interactions (green lines). This brief discussion shows that adsorption was highly dependent on the details of the model used.

It should be stressed that the effect of “superadsorption” at very low densities also occurred for the rigid ligands that did not change their configurations. Thus, it results from a special situation of particles in the confined space near the curved surface between ligands, which act as obstacles. The behavior of particles depends on the resultant of all forces in the systems. In turn, the effective potential is shaped by all pair–pair interactions and changes as the density increases. For a special combination of the system parameters, the effect of “superadsorption” at low densities can be observed. In the case of stronger interactions, this phenomenon does not occur.

**Figure 8:**
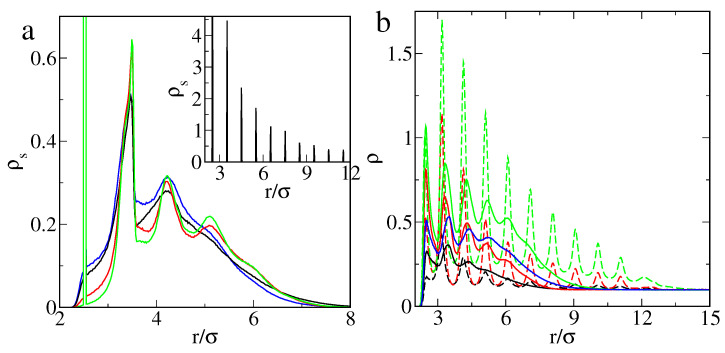
Density profiles of the chain segments (**a**) and fluid particles (**b**) around the core at $\rho_{0}=0.1$ for particles with attached flexible (solid lines) and rigid ligands (dashed lines) plotted for different models and values of $\varepsilon_{Ps}$: models M1 and M2 with 1.5ε (black lines), models M1 and M2 with $\varepsilon_{Ps}=3.0\varepsilon$ (red lines), models M1a and M2a with $\varepsilon_{Ps}=3.0\varepsilon$ (green lines), and model M1a with $\varepsilon_{Ps}=1.5\varepsilon$ (blue line). Other parameters: $\sigma_{c}=4\sigma$, f=20, M=10. In the inset of part a, the segment density profile for the hedgehog-like particle is shown.

Figure 8 presents the density profiles of adsorbed particles and ligand segments at $\rho_{0}=0.1$, where the adsorption is the greatest. To a considerable degree, the structure of the adsorbed fluid replicates the structure of ligands. This was particularly apparent for the hedgehog-like particles (M2). In this case, a series of narrow peaks were observed in the density profiles.

For flexible chains, both the segment density profiles and the fluid density profiles had only a few peaks and decreased continuously in the outer part of the polymer corona. The density of the fluid in the whole surface layer increased for stronger particle–segment interactions and attraction between adsorbed particles. Note that the effect of attraction between particles and between segments was more significant. If these interactions are attractive, the segment density increases not only deep inside the polymer corona but also further from the core.

As mentioned, for flexible tethers, the presence of adsorbed particles influences their configurations. In the framework of the model M1, for stronger particle–segment interactions (compare black and red lines) the segment density was lower near the core, higher for in the middle of the polymer layer, and again slightly higher in the corona periphery. The adsorbed particles push the segments outward from the immediate vicinity of the core. On the other hand, the particles and segments “stick” to each other, allowing the chains to wrap themselves around the particles. The structure of the polymer layer is a result of a complex interplay between these effects.

The attractive interactions between particles and between segments also affected the structure of the polymer layer. For weaker particle–segment interactions ($\varepsilon_{Ps}=1.5\varepsilon$) attraction between particles caused the segment density to increase inside the polymer corona (r<5.5σ) and to decrease outside (black and blue lines). The increase was the strongest in the middle part of the corona. In the case of $\varepsilon_{Ps}=3.0\varepsilon$, the opposite effect was observed in the interior of the brush (red and green lines). However, for 4.5σ<r<5.5σ, the attractions between particles caused an increase of the segment density. It did not influence the behavior of the outer part of the brush.

**Figure 9:**
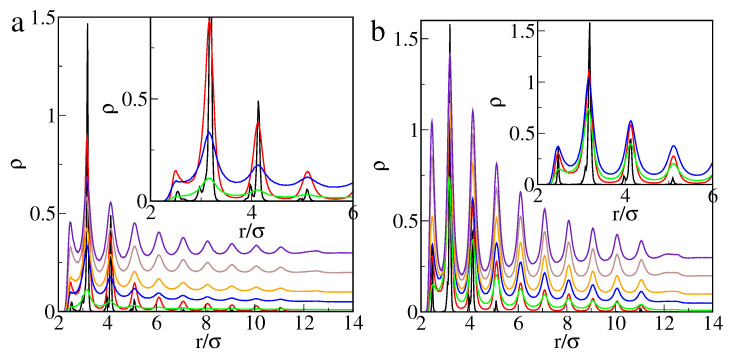
Density profiles of fluid particles around the core plotted for the hedgehog-like particle in the system of the M2-type and $\varepsilon_{Ps}=1.5\varepsilon$ (**a**), 3.0ε (**b**) at different initial densities of the fluid $\rho_{0}=0.0001$ (black), 0.001 (red), 0.01 (green), 0.05 (blue), 0.1 (yellow), 0.2 (brown), and 0.3 (violet). In the insets, the initial parts of the density profiles are shown on a more accurate scale. Other parameters: $\sigma_{c}=4\sigma$, f=20, M=10.

Figure 9 illustrates the “superadsorption” effect at low densities for hedgehog-like particles. In part a, the fluid density profiles obtained for the model M1 with $\varepsilon_{Ps}=1.5\varepsilon$ are shown. A change in the sequence of the profiles plotted for increasing densities $\rho_{0}$ is clearly visible (see inset). If $\varepsilon_{Ps}=3.0\varepsilon$, the profiles for $\rho_{0}=0.001$ (red line) and $\rho_{0}=0.01$ (green line) are intertwined. In the latter case, the excess adsorption monotonically increased in the region of low densities.

**Figure 10:**
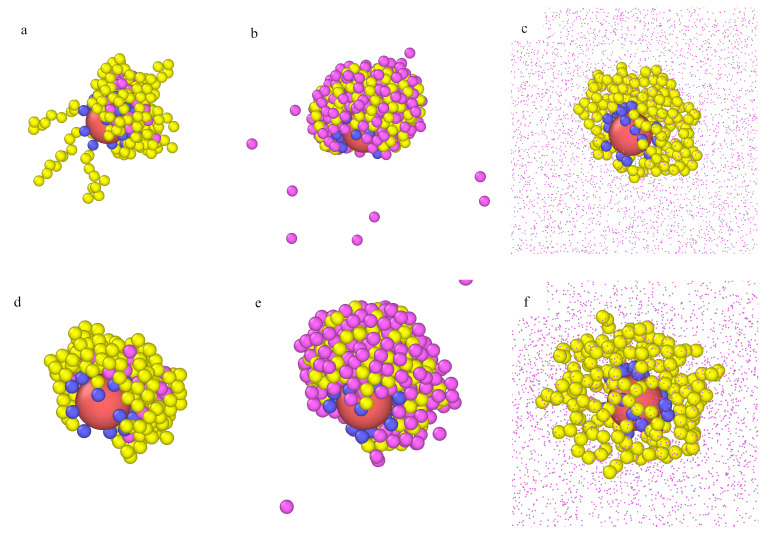
Examples of the equilibrium configurations of hairy particles immersed in fluids of different densities $\rho_{0}=0.0001$ (**a**,**d**), 0.001 (**b**,**e**), 0.1 (**c**,**f**) for model M1 (**a**–**c**), model M1a (**d**–**f**): f=20, M=20 (**a**–**c**), f=20, M=10 (**d**–**f**). Other parameters: $\sigma_{c}=4\sigma$, f=20, M=10 and $\varepsilon_{Ps}=3.0\varepsilon$. The red sphere represents the core, and blue spheres correspond to bonding segments, yellow spheres and pink spheres represent the remaining segments and fluid particles P, respectively. For clarity, particles P are represented by points (size ratios are not kept) in parts (**c**,**f**).

Figure 10 presents selected configurations for the particles with attached flexible ligands. The snapshots for the model M1 and $\varepsilon_{Ps}=3\varepsilon$ for different densities $\rho_{0}$ are shown in the top row. In this case, for $\rho_{0}=0.0001$, the cone-like structure began to be formed. For weaker particle–segment interactions (see Figure 4d–f), the core-shell structure with loosely distributed segments was found at this fluid density. The cone-like structures occurred even for higher densities.

In the bottom row, we present the results for the same particle–segment interactions but for the model M1a. The cone-like structure was observed already at the lowest fluid density. However, at $\rho_{0}=0.1$, the polymer corona became less compact and more symmetrical. Likely, interactions between the adsorbed molecules and molecules in the bulk fluid pull the chains into the surrounding fluid, and the special structure is destroyed. Thus, attractive interactions between fluid molecules considerably affect the structure of hairy particles.

**Figure 11:**
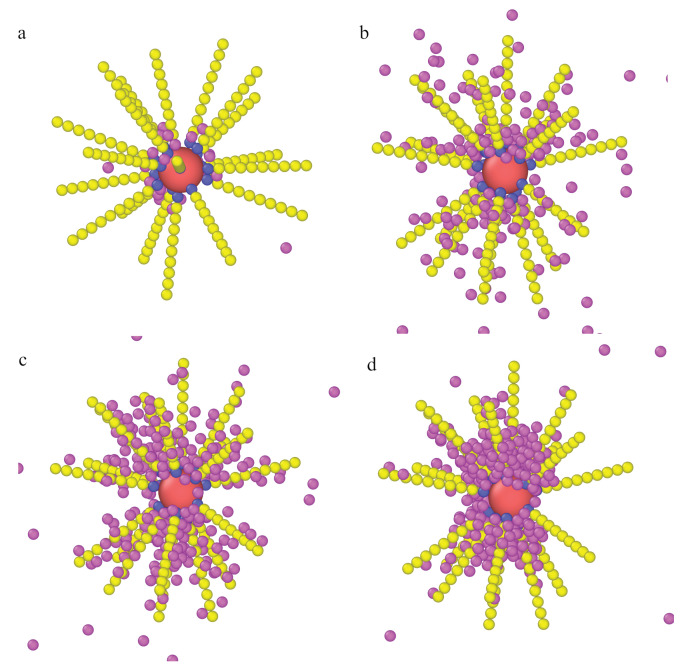
Examples of the equilibrium configurations of the hedgehog-like particles immersed in fluids of densities $\rho_{0}=0.0001$ (**a**) and 0.001 (**b**–**d**) for different models: (**a**,**b**) model M2, $\varepsilon_{Ps}=1.5\varepsilon$, (**c**) model M2, $\varepsilon_{Ps}=3.0\varepsilon$, and (**d**) model M2a, $\varepsilon_{Ps}=3.0\varepsilon$. Other parameters: $\sigma_{c}=4\sigma$, f=20, M=10. The red sphere represents the core, blue spheres correspond to bonding segments, and yellow spheres and pink spheres represent the remaining segments and fluid particles P, respectively.

Finally, we discuss the configurations for the particle with stiff ligands (Figure 11). In this case, the core and ligands form a rigid backbone of the particles and no reconfiguration is possible. In part a, a typical configuration at very low density ($\rho_{0}=0.0001$) is shown. Parts a and b illustrate the behavior of the “basic model” M1. When the density $\rho_{0}$ increased to $\rho_{0}=0.001$, the particles accumulated near the ligands and between them.

The remaining pictures are for this higher density but for different system parameters. If particle–segment attraction was stronger, the adsorption was markedly greater (Figure 11c). The effect of attractive interactions between particles was even more spectacular as seen in comparing configurations (Figure 11b,d). Particles “condense” in the volume between rigid ligands.

In summary, the behavior of nanoparticles with stiff or flexible ligands is significantly different. The details of the models can influence the results of simulations. Nevertheless, the fundamental features of the systems are quite well imitated by the simplest models M1 or M2.

The study showed that the adsorption of particles on hairy particles is a very complex process that depends on many parameters and relations between them. In general, for the system studied, adsorption rose with increasing the grafting density, the length of chains, and the particle–segment interactions.

The general trends observed in our simulations were consistent with previous experimental results [56,57,58,64].

## 4. Conclusions

This work presents the results of coarse-grained simulations for different polymer-functionalized spherical nanoparticles immersed in an explicit fluid consisting of small particles P. We considered the particles with ligands permanently anchored at randomly chosen points at the core surface. We studied nanoparticles with attached flexible chains and rigid ligands.

First, hairy particles modified with perfectly flexible chains were considered. The simplest possible model in which only particle–segment interactions are attractive while the remaining pair interactions are repulsive was discussed. For this model, we demonstrated that, in a wide region of particle densities, the excess adsorption on a hairy nanoparticle increased for longer chains and higher grafting densities. The adsorption properties of hairy nanoparticles of different sizes and flat surfaces modified with grafted chains were compared. When the grafting density was kept constant, the normalized excess adsorption on the flat brush was considerably smaller.

However, the effect of the core size was negligible in a wide region of densities. This was significant only in the case of very low or relatively high bulk densities of adsorbed particles. For the systems with assumed attractive particle–particle and segment–segment interactions, the excess adsorption was greater than in our “basic” model system, where these interactions were repulsive. Our conclusions regarding the adsorption on hairy particles are in line with previous experimental observations [56,57,58].

Adsorption on hairy particles changes the morphology of the polymer corona. Our simulations elucidated the mechanism of adsorption-induced nanoparticle shell reconfiguration of the nanoparticle shell. Adsorbed particles form the bridges between segments belonging both to the same chain and different chains [50,51]. The chains form coils with the particles trapped inside and joined together. We computed the average thickness of polymer coatings for different fluid densities and stated that this is strictly correlated with the excess adsorption isotherms.

We analyzed the dependencies of the brush thickness on the density of adsorbed particles and compared them with the theoretical predictions [19,62,63] and experimental results [56]. Depending on the assumed parameters core-shell or cone-like structures of hairy particles were found in the model systems. In the case of the cone-like particles, the segment cloud was highly asymmetrical. The chains with adsorbed particles accumulated on a part of the core surface, while the remainder was uncovered. The polymer layer could be more or less compact. We found continuous adsorption-induced structural transitions in polymer coatings. The cone-like structure transformed into the core-shell structure at higher densities.

The adsorption-induced local bundling of chains on the nanoparticle surface was observed in the simulations carried out for other systems [48,50] as well as in experiments [56]. In the case of short chains, “the superadsorption” was observed at very low particle densities. The excess adsorption isotherm had a low local maximum at a very low density. For higher densities, however, the excess adsorption isotherm had a typical course. Likely, this follows from the superposition of the effect associated with the confinement of particles near the curved surface between ligands (which act as obstacles) and the effect of the interplay between all interactions in the system. This anomaly occurs only for special combinations of parameters and disappears for stronger attractive interactions.

Finally, we discuss adsorption on the hedgehog-like particles with attached rigid ligands. The adsorption on such particles is higher than on the corresponding hairy particles with flexible chains. If particle–particle interactions are attractive, we observe a “condensation” of fluid in “pores” between ligands.

In summary, we demonstrated how the model parameters affected adsorption on hairy nanoparticles. We analyzed the mechanism of adsorption and the structure of the polymer coating with details. The adsorption on hairy particles follows from competition between interactions near the surface and in the bulk phase and the entropy effects associated with the limiting of possible chain configurations near the core. Hairy particles with flexible chains are “living” adsorbents, which makes the research difficult.

Future studies of adsorption on ligand-tethered nanoparticles can proceed on several fronts. The density functional theory of adsorption on flat brushes [26,27,28,29,33,34] can be adapted to describe the adsorption on hairy nanoparticles. The theoretical predictions can be compared with the results of our simulations. Moreover, the molecular dynamics simulation of adsorption from different explicit solvents will be performed. We hope that our results will help to rationally design hairy particles that could be carriers of bioactive compounds.

## Figures and Tables

**Table 1:** Types of interactions in different models.

Interactions	Models M1, M2	Models M1a, M2a
segment–core	repulsive	repulsive
particle–core	repulsive	repulsive
segment–segment	repulsive	attractive
particle–particle	repulsive	attractive
particle–segment	attractive	attractive
